# Simulated and Experimental Research of Multi-Band Acoustic Metamaterial with a Single Resonant Structure

**DOI:** 10.3390/ma12213469

**Published:** 2019-10-23

**Authors:** Huaijun Chen, Changlin Ding

**Affiliations:** 1 College of Physics and Electronic Information Engineering, Engineering Research Center of Nanostructure and Functional Materials, Ningxia Normal University, Guyuan 756000, China; chenhuaijun79@163.com; 2 Department of Applied Physics, Northwestern Polytechnical University, Xi’an 710129, China

**Keywords:** multi-band acoustic metamaterials, negative modulus, nested split hollow sphere (NSHS), coupling resonance

## Abstract

We present a multi-band acoustic metamaterial (AMM) with a single structural unit of a nested split hollow sphere (NSHS). The transmissions of the NSHS-AMM from the simulation and experiment revealed two dips which were attributed to local coupling resonance. Using the retrieval method from the experimental data, we calculated the effective modulus of the NSHS-AMM and found it to be negative near the bands of the two dips. The AMM with a negative modulus can be easily tuned due to the coupling effect in the NSHS. The two dips can be simultaneously tuned by changing the diameter and the direction angle of the split holes of the interior and exterior split hollow sphere (SHS) in the NSHS. We designed a three-nested SHS-AMM with a negative modulus in three bands. Given the obvious local coupling resonance in the NSHS, such NSHS-AMMs may provide a viable path for the design of broadband AMMs or acoustic metasurfaces.

## 1. Introduction

In the last 20 years, metamaterial research in the field of materials science has undergone rapid development. Although the theory of left-handed materials with simultaneously negative permittivity and negative permeability (electromagnetic metamaterial (EMM)) has been presented since 1968 [1], the development of this field has been limited because finding a material with negative permeability in nature is difficult. In 1999, Pendry presented artificial split resonant rings (SRRs) with magnetic resonance [2], and a double-negative EMM with negative refraction was later fabricated [3,4]. EMMs and electromagnetic metasurfaces (EMSs) have since been designed with several unique experimental effects, such as flat focusing, subwavelength imaging, cloaking, trapped rainbow effect, polarisation conversion, and optical vortex [5,6,7,8,9,10,11]. Acoustic metamaterials (AMMs) and acoustic metasurfaces (AMSs) are designed using the analogy method based on the concept of local resonance [12,13] to manipulate acoustic waves in a distinct manner. AMMs are characterised by double-negative parameters, negative refraction, cloaking, perfect sound absorption, inverse Doppler Effect, and others [14,15,16,17,18,19,20]. AMSs with sub-wavelength thickness follow the generalised Snell law for acoustic waves; hence, acoustic waves may, in theory, be manipulated at will by AMSs. [21,22,23,24,25,26]. Our group presented a split hollow sphere (SHS) and split tube (ST) based on the Helmholtz resonator model to achieve an AMM with a negative modulus and/or negative mass density [27,28,29]. We also systematically investigated the unusual properties of AMMs. The SHS and ST structure can be used to achieve AMSs with anomalous reflection and refraction [23,24,25].

Nevertheless, the research on AMMs and AMSs based on local resonance remains limited because AMMs and AMSs only have an abnormal response near resonant frequencies. When they are far from the resonant band, the unusual property disappears. Therefore, some researchers have attempted to design broadband AMMs or AMSs. The main method has been to assemble several types of acoustic atoms with local resonance, which may produce resonant coupling and overlap with multiple resonant bands to realise a broadband response [30,31,32,33,34]. Huang et al. utilised a dissipative multi-resonator to obtain broadband elastic wave attenuation [30,31]. Ding et al. combined SHSs with different split holes to achieve multi-band and broadband AMMs with a negative modulus [33]. Such methods require several resonators with weak interactions, and that each unit cell exert its resonant function independently. Furthermore, a broadband negative mass density response could be realised by controlling the anisotropy of materials based on the intrinsic properties of unit cells [35,36].

In the current study, we presented a single structural unit of a nested split hollow sphere (NSHS), that can realise AMM with a negative modulus in two bands. The NSHS structure consists of two resonant atoms of the SHSs and featured mutual coupling to manipulate the resonance of the NSHS. We investigated the relationship between resonant dips and the internal geometry size of the NSHS structure to explore a suitable method for fabricating broadband AMMs and AMSs.

## 2. Model Analysis and Experimental Setup

**Figure 1:**
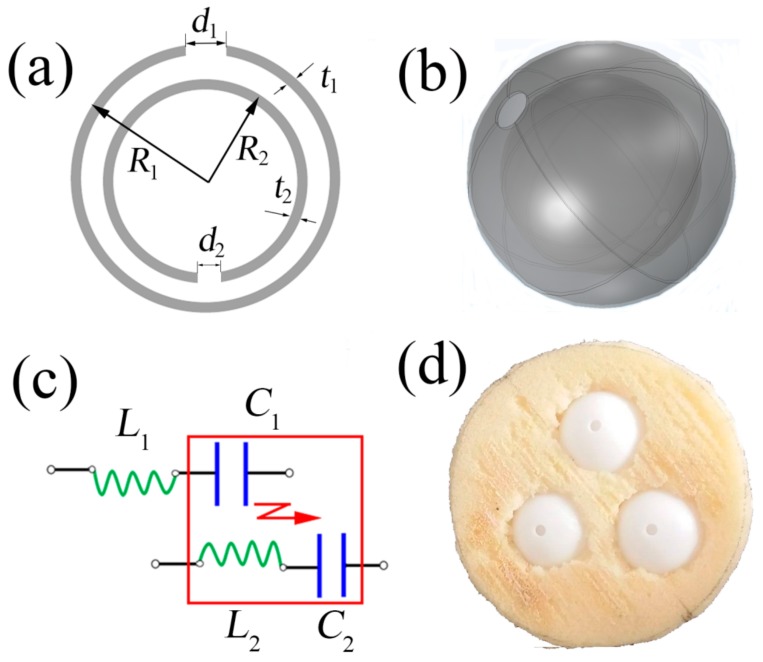
(**a**) 2D and (**b**) 3D schematic of the nested split hollow sphere (NSHS) structure, (**c**) effective acoustic inductor–capacitor (L–C) circuit and (**d**) NSHS-multi-band acoustic metamaterial (AMM) image.

SRR is a kind of magnetic resonance structure for designing EMMs. Considering that AMMs are analogous to EMMs, we designed an NSHS structure with the same 2D schematic as SRRs (Figure 1a). The NSHS structure was an acoustic resonance structure composed of two SHSs with the same ball centre, as shown in Figure 1b. *R*_1_, *t*_1_ and *d*_1_ are the radius, thickness and split hole diameter of the exterior SHS, respectively. *R*_2_, *t*_2_ and *d*_2_ are the radius, thickness and split hole diameter of the interior SHS, respectively. The NSHS was 3D printed using a photosensitive resin (PSR) with a thickness of 0.5 mm. As the acoustic impedance of PSR is much larger than that of air, the sphere shell could be considered an acoustically rigid medium. According to the lumped circuit model, the NSHS microstructure could be analogous to two inductor–capacitor (L–C) circuits with mutual inductance, as shown in Figure 1c. The hollow sphere cavities of two SHSs function as acoustic capacitors, that is,

(1) $$C_{1}=V_{1}/(\rho_{0}c_{0}^{2})$$

(2) $$C_{2}=V_{2}/(\rho_{0}c_{0}^{2})$$

The two split holes serve as acoustic inductances, that is,

(3) $$L_{1}=\rho_{0}t\prime /S_{1}$$

(4) $$L_{2}=\rho_{0}t^{\prime \prime }/S_{2}$$

where *V*_1_ and *V*_2_ are the effective volumes of the hollow spheres, *ρ*_0_ is the density of air, *c*_0_ is the sound speed in air and $S_{1}=\frac{1}{4}\pi d_{1}^{2}$ and $S_{2}=\frac{1}{4}\pi d_{2}^{2}$ are the cross-section sizes of the two split holes. t′ and $t^{\prime \prime }$ are the effective lengths of the exterior and interior split holes in the NSHS, respectively. According to Ref. [12], the air oscillation from the split holes of the NSHS will increase their effective lengths as

(5) $$t\prime =t_{1}+\delta_{1}\sqrt{d_{1}}$$

(6) $$t^{\prime \prime }=t_{2}+\delta_{2}\sqrt{d_{2}}$$

where $\delta_{1}$ and $\delta_{2}$ are the coupling coefficient between the two SHSs in the NSHS. From the resonant model, the two existing resonant frequencies in the NSHS are

(7) $$f_{1}=1/(2\pi \sqrt{L_{1}C_{1}})$$

(8) $$f_{2}=1/(2\pi \sqrt{L_{2}C_{2}})$$

A full-wave simulation was conducted to investigate the NSHS-AMM by using COMSOL Multiphysics software (Version 5.3a, COMSOL Inc., Stockholm, Sweden) based on the finite element method. The simulation environment was a rectangular acoustic waveguide filled with air via the acoustic–thermoacoustic interaction module in COMSOL. The NSHS was placed in a waveguide with a periodic boundary to calculate the acoustic properties of the AMM with periodic NSHS array. The left and right-side faces were set as radiation boundaries. A plane harmonic acoustic wave (1 Pa) was perpendicularly incident on the left-side face. The cavities of the hollow sphere and split holes were filled with air and set as the thermoacoustic domain. The sound speed, density and bulk viscosity of air were set as *c*_0_ = 343 m/s, *ρ*_0_ = 1.29 kg/m^3^ and *μ*_b_ = 3.08 × 10^−3^ Pa s, respectively. The entire domain of the waveguide was meshed by free tetrahedrons based on face meshing, and the user predefined size was set to ‘refine’, implying that the maximum element size was 9.6 mm, the minimum element size was 0.6 mm and the maximum element growth rate was 1.4 mm. The model was calculated in the frequency domain, and the solvers were chosen as ‘MUMPS’.

**Figure 2:**
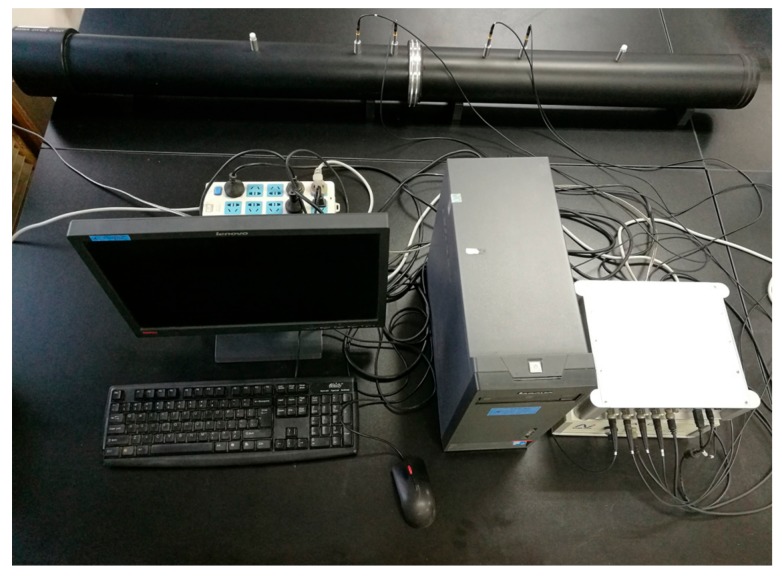
Photograph of the impedance tube testing system.

A sponge matrix with a diameter of 100 mm and a thickness of 40 mm was used to fabricate the NSHS-AMM in the experiment. The unit cell of the NSHS with a lattice constant of 45 mm was arranged in the sponge matrix to fabricate the NSHS-AMM. Figure 1d presents the physical image of the sample. The sphere radii of the NSHS were *R*_1_ = 20 mm and *R*_2_ = 15 mm, and the diameters of the two split holes were *d*_1_ = 6 mm and *d*_2_ = 3 mm. Two kinds of SHS-AMMs were fabricated by filling the SHS structures instead of the NSHSs to investigate the transmission characteristics and compare them with the transmitted results of the NSHS-AMM. The first SHS-AMM consisted of three SHSs with the same size as the internal SHS of the NSHS. The second SHS-AMM was composed of the three external SHSs of the NSHS. The two different sized SHS structures were designed with the same resonant frequency according to Equation (7). When the two SHS structures are used to design NSHS, the two shifted transmission dips will clearly illustrate the characteristics of coupling resonance. All samples were measured in an impedance tube testing system. The testing equipment (Figure 2) consisted of an impedance tube which had a diameter of 100 mm, four microphones, a power amplifier, a data-collecting analyser, a computer, and some data cables.

## 3. Results and Discussion

### 3.1. Dual-Band AMM

**Figure 3:**
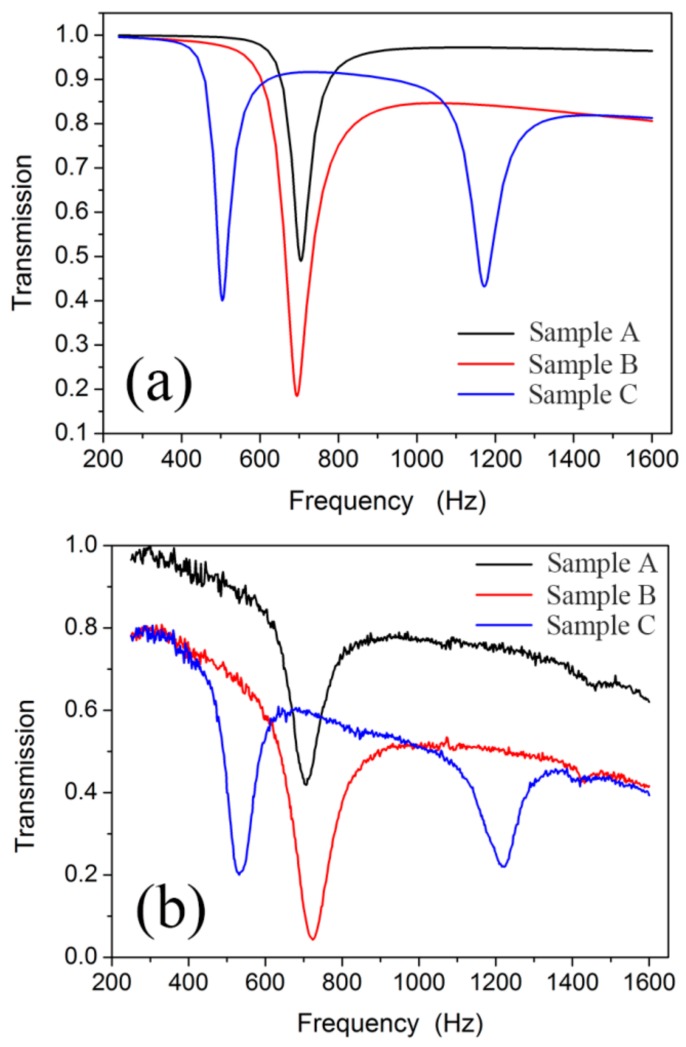
The simulated (**a**) and experimental (**b**) transmission curves of the first SHS-AMM (sample A), second SHS-AMM (sample B) and the NSHS-AMM (sample C).

The simulated transmission results of the NSHS-AMM and two SHS-AMMs are shown in Figure 3a. Each SHS-AMM only presents one transmission dip near the resonant frequency of 700 Hz because of the weak interaction between the adjacent SHSs [13,33]. By contrast, the NSHS-AMM presents two transmission dips at 504 and 1172 Hz. In Figure 3b, the experimental transmission curve of the NSHS-AMM shows two dips located in the frequency of 532 and 1222 Hz. Each SHS-AMM presents a transmission dip near 700 Hz. All transmission results of the three samples in the experiment are in good agreement with the simulation results. The difference between the simulation and the experiment is attributable to machining errors. The transmissions in Figure 3 were normalized by the ratio of transmitted sound to incident sound in the experiment and the simulation. As the thermal viscosity loss of the acoustic medial increases with increasing frequency, there will be more sound absorption in the higher frequency which will lead to decreased transmissions of samples in the non-resonant frequency region.

**Figure 4:**
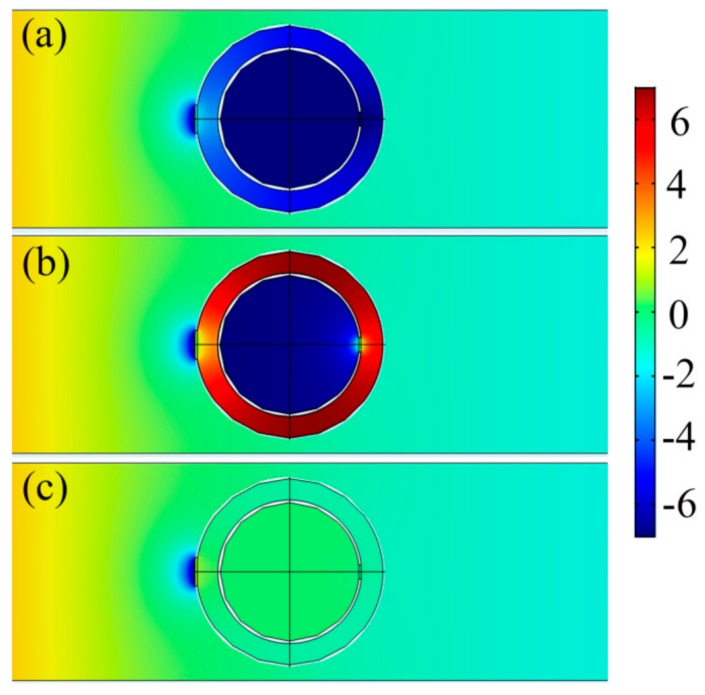
The acoustic pressure field distribution of the NSHS-AMM. (**a**) 504 Hz; (**b**) 1172 Hz; (**c**) 1900 Hz.

Although the NSHS consists of the two resonant structures of the SHS samples, the two dips are not in the resonant bands of the two SHSs. We calculated the acoustic field distribution in the waveguide to illustrate the problem and present the results in Figure 4. In the frequencies of the two dips (504 and 1172 Hz), the NSHS can store strong acoustic energy and release it from the two split holes, which means resonance in the structure. Figure 4a indicates that the maximum negative pressure is distributed into the interior cavity in the first dip, representing the resonance of the interior SHS. Figure 4b shows that the maximum positive pressure is distributed into the exterior cavity in the second dip, representing the resonance of the exterior SHS. In the non-resonant frequency of the NSHS (1900 Hz), the pressure is nearly equally distributed throughout the entire domain, as shown in Figure 4c. Obviously, this condition is attributed to the coupling effect in the NSHS structure which can affect the resonant frequency band. The coupling in the NSHS results in the redshift of the interior resonance. The exterior sphere shell causes the released energy to oscillate back and forth between the interior split hole and the shell, thereby extending the effective length $t^{\prime \prime }$ of the interior SHS. Equations (4) and (6) show that the resonant frequency of the interior SHS can shift to a low frequency. As the interior SHS decreases the effective cavity volume of the exterior SHS, the resonant frequency of the exterior SHS may also cause a blue shift, as shown in Equations (1) and (7).

In the experiment, we retrieved the effective parameters of NSHS-AMMs. According to the homogeneous media theory, the acoustic refractive index *n* and impedance *Z*_eff_ can be expressed as follows by inverse processing:

(9) $$n=\pm \;\frac{1}{kL}\cos^{-1}[\frac{1}{2T}(1-R^{2}+T^{2})]+\frac{2\pi m}{kL}$$

(10) $$Z_{\mathrm{eff}}=\pm \sqrt{\frac{{\left(1+R\right)}^{2}-T^{2}}{{\left(1-R\right)}^{2}-T^{2}}}$$

where *m* is the branch number of the cos^−1^ function. The *T* is the transmission coefficient and *R* is the coefficient. The *k* is the wave vector of acoustic waves; *L* is the effective thickness of the AMM. Then, the effective modulus and mass density can be expressed as

(11) $$E_{\mathrm{eff}}=\frac{Z_{\mathrm{eff}}}{n}E_{0}$$

(12) $$\rho_{\mathrm{eff}}=nZ_{\mathrm{eff}}\rho_{0}$$

**Figure 5:**
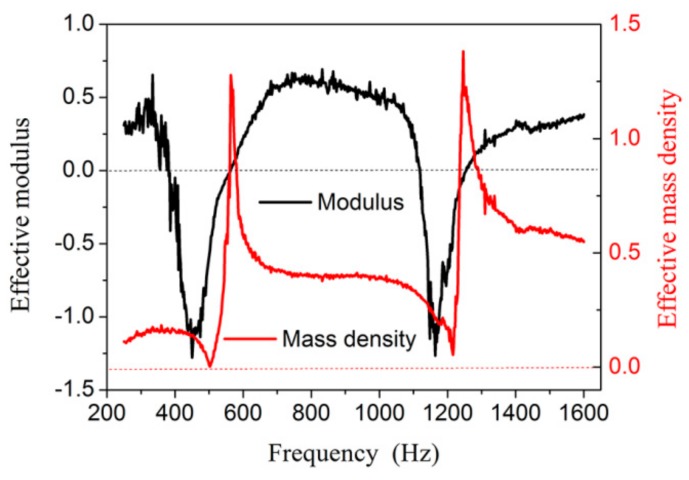
The calculated effective acoustic parameters of NSHS-AMM (the red curve is the effective mass density and the black curve is the effective modulus).

Then, we can calculate the effective mass density and modulus of the NSHS-AMMs from the experimental transmission and the reflection coefficients (Figure 5). The NSHS-AMM presents two bands with a negative modulus near the resonant frequency, and the mass density is always positive. Therefore, the NSHS-AMM is a type of dual-band AMM with a negative modulus.

### 3.2. Tuneable Dual-Band AMM via Geometry Structure

#### 3.2.1. Directional Angle

**Figure 6:**
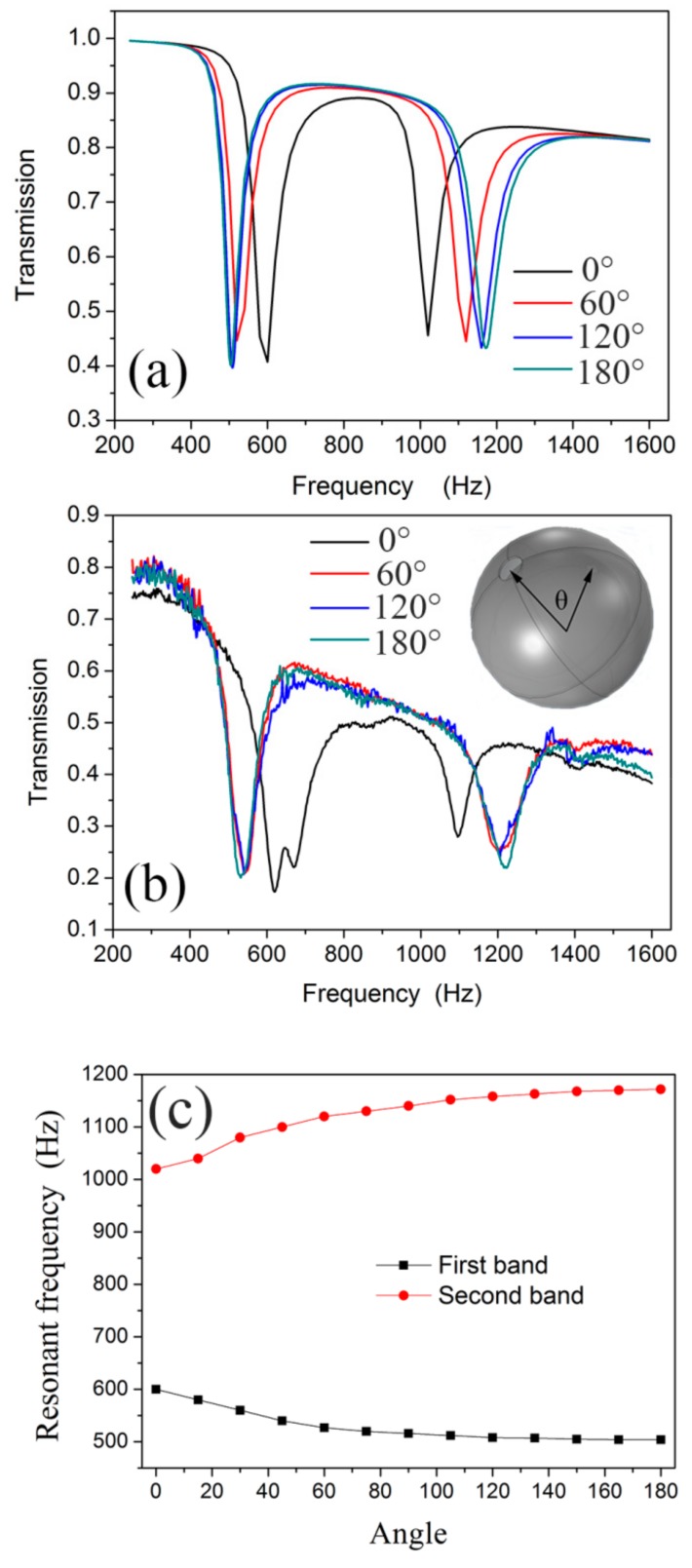
(**a**) The simulated and (**b**) experimental transmission with the directional angle *θ* of the split holes for NSHS-AMM; (**c**) the relation between the simulated dips frequencies and directional angle *θ.*

Given the complex coupling of the two sub-resonators in the NSHS, the NSHS-AMM with a dual negative modulus band exhibits abundant tuneable properties. The inset in the upper right corner of Figure 6b shows the directional angle *θ* between the two split holes in the NSHS. The experimental sample discussed in Section 3.1 has the angle *θ* of 180°. When the directional angle *θ* increases from 0° to 180° with an interval of 60°, the four NSHS-AMMs present two transmission dips in the simulation and the experiment, as shown in Figure 6a,b, respectively. It is noted that the NSHS-AMM with a 0° angle presented a fluctuation in the first dip of 600–700 Hz, which is attributed to the machining error. When one NSHS with a 0° degree is machined with deviation, its resonant frequency will shift to another band. As there is weak interaction between the units, the sample will present two transmission dips as a fluctuation in the first dip band. The simulated and experimental results agree well with each other. We also simulated the dip frequency distribution of the sample with *θ* span of 15° (Figure 6c). The figure shows that the two dips had negligible frequency shifts because angle *θ* was larger than 60°. However, the two dips evidently shifted when angle *θ* was lower than 60°. All the dip depths were similar to one another. The two split holes in the NSHS with an adequately large distance exerted a negligible effect on the coupling resonance of the AMM. However, when the two split holes were close to each other, the coupling effect evidently produced dual resonant frequency shifts (the first dip moves to the high frequency, and the second dip moves to the low frequency). Therefore, we attribute the simulated and experimental results to the oscillation coupling of the released energy from the two split holes. When the two split holes were close to each other, the oscillation coupling of the released energy from the two split holes was strong.

#### 3.2.2. Diameter of the Exterior Split Hole

**Figure 7:**
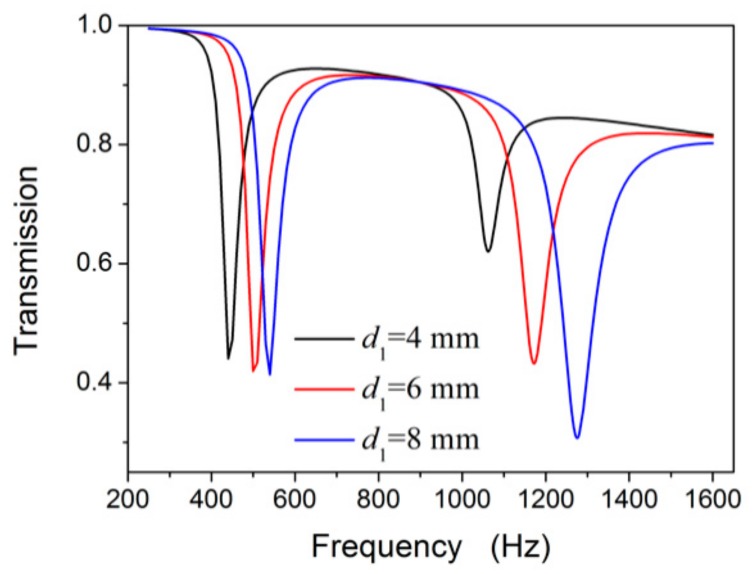
The transmission curves of NSHS-AMM with different diameters of the external split hole.

Given that the simulated results are in accordance with the experimental results (Section 3.1 and Section 3.2.1), we investigated the results via simulation. We first studied the effects of the diameter of the external split hole *d*_1_ to further investigate the coupling effect and the tuneable transmission dips of the NSHS-AMM with different geometry sizes. The other parameters were set to be the same as those in Section 2. Figure 7 shows the transmission results of the three types of NSHS-AMM with *d*_1_ of 4, 6 and 8 mm. The three NSHS-AMMs show two transmission dips which move to high frequencies as the parameter *d*_1_ increases. The frequency shift range of the first dip is less than that of the second dip. This also indicates that the depth of the second dip becomes deeper, whereas the depth of the first dip shows a negligible change as the parameter *d*_1_ increases. The results illustrate that *d*_1_ mainly affected the frequency band of the second dip because of the resonance of the external SHS. As the increased *d*_1_ adds more released energy to oscillate in and out of the cavity, the resonance of the external sub-resonator can be enhanced, and the transmission dip deepens further.

#### 3.2.3. Diameter of the Interior Split Hole

**Figure 8:**
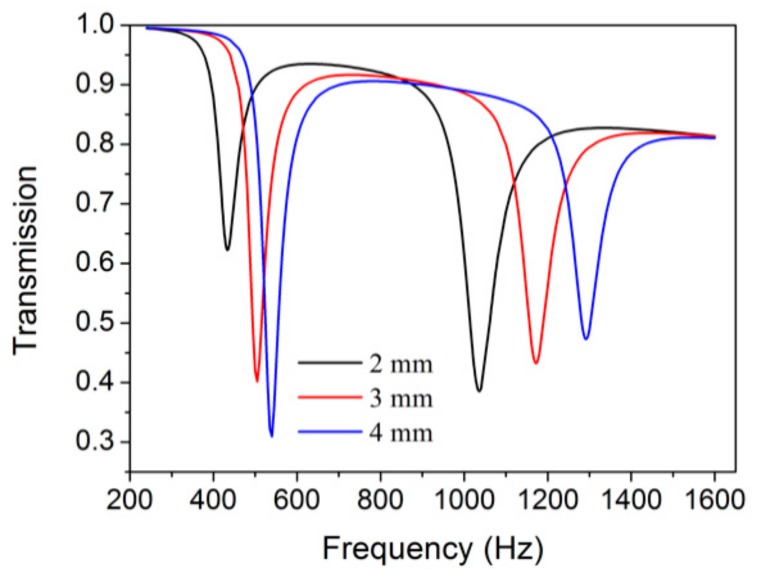
The transmission curves of NSHS-AMM with different diameters of the interior split hole.

When the *d*_2_ of the split holes in the internal sub-resonator increased from 2 mm to 4 mm, the NSHS-AMMs still showed two transmission dips. With the increase of *d*_2_, the first dip and second dip shifted to a high frequency, but with the latter showing greater movement. Thus, the large internal split holes can tune the resonance of the two sub-resonators in the NSHS to a high frequency band. The depth of the transmission dips is indicative of resonance strength. Figure 8 shows that the resonance of the first dip is strong and that of the second dip is weak. The strength of the first dip is determined by the internal SHS in which the increased *d*_2_ enhances the released energy from the split hole. The weak resonance of the second dip is due to the resonance of the external SHS in which the increased *d*_2_ may weaken the stored energy in the cavity.

### 3.3. Multi-Band AMM

**Figure 9:**
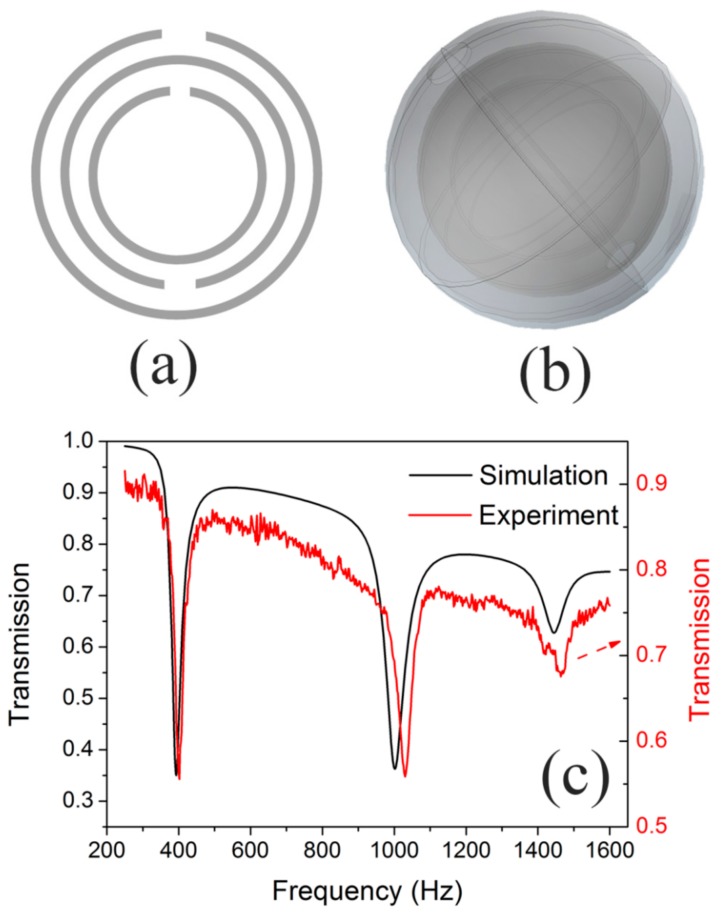
(**a**) the 2D and (**b**) 3D schematic diagram of TNSHS structure, (**c**) the transmission results of the NSHS-AMM.

According to the results in Section 3.2, the geometry size of the NSHS affect resonant coupling and the transmission dips. We further designed a structure of a three-nested SHS (TNSHS), as shown in Figure 9a,b. To fabricate the TNSHS structure in an experiment by the 3D printing method, the interior two SHSs were designed from the structure of the NSHS, as discussed in Section 3.1. In this structure, we only changed the split hole’s diameter *d*_2_ to 5 mm and the thickness of sphere *t*_1_ and *t*_2_ to 0.7 mm. The exterior SHS had a sphere diameter of 50 mm, a sphere shell thickness of 1 mm and a split hole diameter of 10 mm. The directional angle between the adjacent split holes was 180°. Using the simulated method in Section 2, we calculated the transmission curve, which is the black curve shown in Figure 9c. The NSHS-AMM show three transmission dips related to the resonance of the three sub-resonators of the SHSs. From Figure 9c, the red curve is the experimental transmission curve, which agrees very well with the result in simulation. The three dips indicate the three bands with negative moduli. This kind of NSHS-AMM has a negative modulus in three bands. If we were to design an *n*-nested SHS structure (*n* may be equal to four or more), the *n*-band AMM with a negative modulus could be fabricated with only one type of structure. This method gives us another choice for designing broadband AMMs.

## 4. Conclusions

We presented an NSHS-AMM with dual- and multi-band negative moduli via simulations and experiments. The relationship between the resonant dips and the internal geometry size of the NSHS structure was investigated. The findings show that the coupling between the two split holes can cause the first dip to move to a high frequency and the second dip to move to a low frequency. The large-sized split holes can produce a high resonant band for the two transmission dips because of the coupling effect between the two sub-resonators in the NSHS. On the basis of our study of the NSHS, we further designed a TNSHS structure with three sub-resonators. The simulation illustrates that the NSHS-AMM presented three transmission dips with negative moduli. In theory, we could fabricate a multi-band AMM with extensive transmission dips. This type of multi-band AMM with a single structure could provide a potential path for achieving broadband AMMs or AMSs.
